# The Challenges of Dialysis in Systemic Sclerosis: Between the Devil and the Deep Blue Sea?

**DOI:** 10.1155/2012/865193

**Published:** 2012-09-10

**Authors:** N. Brown, A. Summers, M. C. Venning, I. N. Bruce

**Affiliations:** ^1^Renal Unit, Manchester Royal Infirmary, Central Manchester Foundation Trust, Oxford Road, Manchester M13 9WL, UK; ^2^Arthritis Research Unit, University of Manchester, Oxford Road, Manchester M13 9PL, UK

## Abstract

We present the case of a patient with systemic sclerosis (SSc) and end stage renal disease (ESRD) who experienced complications of both peritoneal and haemodialysis. We review previously reported outcomes of patients with systemic sclerosis on dialysis and discuss potential shared mechanisms in both the disease pathogenesis and dialysis-related complications, particularly with regards to encapsulating peritoneal sclerosis (EPS).

## 1. Introduction

Systemic sclerosis (SSc) is a rare but important cause of end-stage renal disease (ESRD). We present a case which outlines dialysis complications which may potentially arise as a consequence of the underlying disease. This case highlights the challenges which may be faced by the patient and clinician in choosing the optimum modality of renal replacement therapy (RRT) for those with systemic sclerosis.

## 2. Case Report

We describe the case of a 51-year-old female who presented early in 2003 with a polyarthritis. She was initially thought to have rheumatoid arthritis and was treated with intramuscular triamcinolone and hydroxychloroquine. This was subsequently discontinued due to side effects of vomiting and diarrhoea. She presented shortly following this to a district General Hospital with renal failure, malignant hypertension, and microangiopathy. During her acute admission, she developed pulmonary oedema and seizures requiring intubation, intensive care admission, and haemofiltration. Renal biopsy was contraindicated at that time due to low platelets and bleeding risk. Due to nonrecovery of renal function, she was transferred to our renal unit for Tenckhoff catheter insertion and commenced automated peritoneal dialysis.

During this admission, she was reviewed by the Rheumatology team who noted a history of Raynaud's phenomenon as well as the polyarthropathy and commented on some tight skin over the distal phalanges. Her immunological tests demonstrated a positive antinuclear antibody, titre 1 : 300, with both a speckled and nucleolar pattern. However, double-stranded DNA antibody was negative, and complement was normal. In view of her clinical and immunological findings, it was felt that her microangiopathy was related to an underlying connective tissue disorder, potentially a lupus/systemic sclerosis overlap. She started on low-dose prednisolone to which her musculoskeletal symptoms responded and was subsequently maintained on 5mg prednisolone a day. Following the acute presentation, she started on an angiotensin converting enzyme inhibitor and blood pressure came under control.

**Figure 1:**
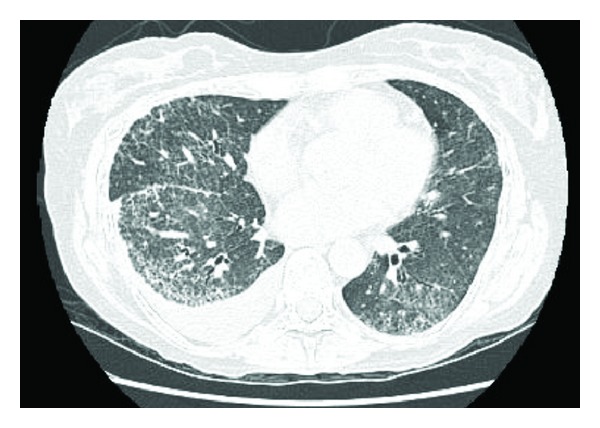
HRCT showing UIP (followup scan, May 2008).

Under followup over the next 2 years she developed more pronounced sclerodactyly and gastric antral vascular Ectasia (or watermelon stomach) with these clinical features confirming her diagnosis of systemic sclerosis. She became transiently positive for lupus anticoagulant in 2005, but this was negative on repeat testing along with negative anticardiolipin antibodies and no thrombotic events. She remained fairly stable, though dialysis dependent, for 4 years with the only additional treatment required for her connective tissue disorder being annual epoprostenol infusions for her Raynaud's phenomena. In 2007, she was noted to have interstitial lung disease on high resolution computer tomography (CT) thorax, which showed evidence of widespread usual interstitial pneumonia (UIP) type pulmonary fibrosis with fine reticulation and traction bronchiectasis (Figure 1).

She was subsequently initiated on mycophenolate mofetil as a treatment for her interstitial lung disease and remained relatively stable from a respiratory point of view. She underwent a CT of her abdomen in September 2008 as part of a “CT surveillance programme” in place at that time for those who had been on peritoneal dialysis (PD) for more than 5 years. This showed no evidence of peritoneal thickening or calcification. PD adequacy testing showed Kt/V of 3.35/week (target > 1.3) with a high transporter membrane status. However, in late 2009 she was admitted with diarrhoea and a displaced PD catheter. She was temporarily transferred to haemodialysis and took the decision to continue with haemodialysis as her modality of renal replacement therapy. She had been on PD for a total of 64 months. During this time, she had only a single episode of culture-proven peritonitis.

She was referred for left brachiocephalic fistula formation, but unfortunately this failed with thrombosis in the median cubital vein. Therefore, haemodialysis was continued via a dual-lumen-tunnelled dialysis catheter. Antiphospholipid antibody tests were not repeated at this time point.

**Figure 2:**
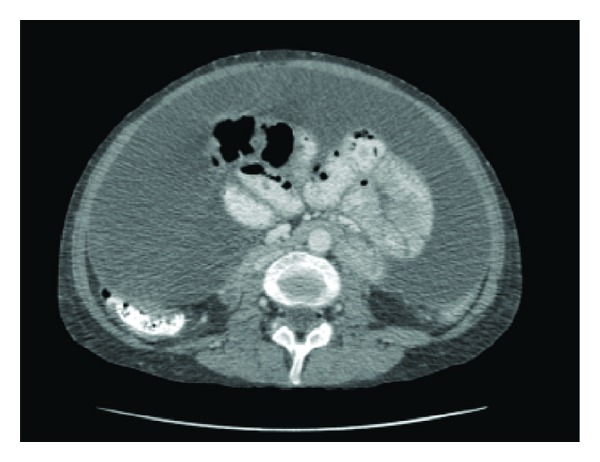
CT abdomen showing ascites and thickened peritoneum (June 2010).

In May 2010, she was diagnosed with breast cancer (multifocal invasive ductal carcinoma) and underwent a total mastectomy and lymph node clearance. In June 2010, she was admitted with vomiting, weight loss (>10% body mass), and abdominal tenderness. CT abdomen showed thickening and enhancement of the peritoneal surfaces and the presence of septated ascites. (Figure 2). Ascitic fluid showed no evidence of malignant cells. Due to the above combination of clinical and radiological findings, a diagnosis of encapsulating peritoneal sclerosis (EPS) was made. This was confirmed at the time of peritonectomy with the macroscopic finding of the typical abdominal cocoon as the signature of EPS caused by long-term peritoneal dialysis. The surgical intervention successfully alleviated the bowel obstruction, and no postoperative complications occurred.

A further attempt at arteriovenous fistula formation was unsuccessful, and dialysis was continued via a catheter. In spite of treatment with radiotherapy and tamoxifen her cancer progressed with multiple liver, splenic, and bone metastases presented April 2011. Following commencement of chemotherapy in May 2011, she was admitted with neutropenia and pneumonia and died shortly following admission.

We present this case to highlight the difficulties faced when choosing a dialysis modality for patients with SSc due to the potential impact of this disease upon the success of dialytic therapies.

## 3. Discussion

We present a woman with connective tissue disease and a seemingly progressive systemic sclerotic phenotype. This was characterised by Raynaud's phenomenon, sclerodactyly, and facial telangectasia, with late development of watermelon stomach and pulmonary fibrosis.

It has long been recognized that the most serious complication of peritoneal dialysis is the development of encapsulating peritoneal sclerosis (EPS). It is characterised by a progressive, intra-abdominal, inflammatory process resulting in sheets of fibrous tissue which cover, bind, and constrict the viscera, thereby compromising the motility and function of the bowel. At the molecular level, there are many common fibrotic and angiogenic factors (TGF, VEGF) which have been implicated in the pathogenesis of systemic sclerosis and EPS [1, 2]. It is therefore possible that patients with systemic sclerosis are more likely to develop multiorgan fibrosis, including the spectrum of EPS. However, to our knowledge, this is the first report of any possible association between EPS and systemic sclerosis. Moreover, the question of technique survival in PD patients with this sclerotic phenotype still needs investigation.

A recent publication reviewed dialysis outcomes of 127 SSc patients in the Australia/New Zealand (ANZDATA) dialysis registry [3]. 50% of these patients were treated with PD reflecting the much higher use of this dialysis modality in these countries. Incidence of EPS was not specifically reported in this population. Cause of death was primarily from cardiovascular disease followed by withdrawal from dialysis, which may indicate peritoneal dialysis failure. Median survival on dialysis in this population was only 2.43 years, perhaps not providing long enough exposure to peritoneal dialysis to develop the complication of EPS.

This case of a patient with systemic sclerosis and EPS, yet without any of the conventional risk factors (e.g., recurrent peritonitis, excessive PD vintage), raises the question as to whether systemic sclerosis itself may predispose to EPS. Similarly, the failure of two fistula attempts is likely to represent a diminished fistula success chance in patients with the vasculopathy of SSc, possibly in part due to circulating profibrotic and angiogenic molecules [4]. It has also been suggested that the presence of antiphospholipid antibodies may play a part in the pathogenesis of vascular events in SSc [5].

Systemic sclerosis patients have impaired flow-mediated vasodilatation, reflecting abnormal endothelial function [6]. There is no data reporting the outcomes of dialysis fistula formation in this population, but events due to atherosclerotic and structural vascular disease are well described in patients with SSc [7].

In summary, further data is required regarding outcomes on dialysis for patients with systemic sclerosis, specifically with regards to vascular access outcomes, peritoneal membrane failure, and development of EPS. This will allow both clinicians and patients to be better informed when making decisions concerning the optimum mode of dialysis in this population.
